# Supplementary material for: Patterns of COVID-19 testing and mortality by race and ethnicity among United States veterans: A nationwide cohort study
Source: PLoS Med. 2020 Sep 22;17(9):e1003379. doi: 10.1371/journal.pmed.1003379 (PMC7508372; doi:10.1371/journal.pmed.1003379)
Supplement: S2 Table — (DOCX) [file pmed.1003379.s003.docx]

| **Table S2.** Individual adjustments for the association between race/ethnicity and Covid-19 positivity and mortality | | | | | | | | | |
| --- | --- | --- | --- | --- | --- | --- | --- | --- | --- |
|  | **Positive test result among tested** | | | |  | **30-day mortality among cases** | | | |
|  | **Black vs White** | | **Hispanic vs White** | |  | **Black vs White** | | **Hispanic vs White** | |
|  | **OR (95% CI)** | **p** | **OR (95% CI)** | **p** |  | **OR (95% CI)** | **p** | **OR (95% CI)** | **p** |
| **Unadjusted** | 2.49 (2.40-2.58) | <.0001 | 2.80 (2.67-2.94) | <.0001 |  | 0.76 (0.66-0.88) | 0.0002 | 0.60 (0.47-0.77) | <.0001 |
| **Age-adjusted** | 2.48 (2.40-2.57) | <.0001 | 2.56 (2.44-2.69) | <.0001 |  | 1.08 (0.93-1.26) | 0.32 | 1.11 (0.85-1.44) | 0.46 |
| +Sex | 2.52 (2.43-2.61) | <.0001 | 2.56 (2.44-2.68) | <.0001 |  | 1.08 (0.93-1.26) | 0.32 | 1.10 (0.84-1.43) | 0.50 |
| +Rural/urban residence | 2.27 (2.19-2.36) | <.0001 | 2.34 (2.23-2.46) | <.0001 |  | 1.07 (0.91-1.25) | 0.41 | 1.09 (0.83-1.43) | 0.52 |
| +Site of care | 1.98 (1.90-2.06) | <.0001 | 2.02 (1.91-2.13) | <.0001 |  | 1.02 (0.85-1.23) | 0.83 | 0.98 (0.73-1.33) | 0.92 |
| **Demographic-adjusted** | 1.92 (1.85-2.00) | <.0001 | 1.96 (1.86-2.07) | <.0001 |  | 1.02 (0.84-1.22) | 0.87 | 0.98 (0.72-1.31) | 0.87 |
| +Asthma | 1.93 (1.85-2.01) | <.0001 | 1.97 (1.86-2.08) | <.0001 |  | 1.01 (0.84-1.22) | 0.89 | 0.97 (0.72-1.31) | 0.85 |
| +Cancer | 1.94 (1.86-2.02) | <.0001 | 1.95 (1.85-2.06) | <.0001 |  | 1.01 (0.84-1.22) | 0.90 | 0.98 (0.73-1.32) | 0.89 |
| +Chronic kidney disease | 1.93 (1.85-2.01) | <.0001 | 1.97 (1.86-2.08) | <.0001 |  | 0.97 (0.80-1.17) | 0.73 | 0.98 (0.72-1.32) | 0.87 |
| +COPD | 1.90 (1.83-1.98) | <.0001 | 1.94 (1.83-2.04) | <.0001 |  | 1.03 (0.86-1.24) | 0.74 | 1.00 (0.74-1.34) | 0.98 |
| +Diabetes mellitus | 1.91 (1.83-1.99) | <.0001 | 1.95 (1.84-2.06) | <.0001 |  | 1.00 (0.83-1.20) | 0.96 | 0.96 (0.71-1.30) | 0.80 |
| +Hypertension | 1.94 (1.86-2.02) | <.0001 | 1.97 (1.86-2.08) | <.0001 |  | 0.99 (0.82-1.19) | 0.92 | 0.97 (0.72-1.31) | 0.84 |
| +Liver disease | 1.93 (1.85-2.01) | <.0001 | 1.97 (1.87-2.08) | <.0001 |  | 1.01 (0.84-1.21) | 0.94 | 0.97 (0.72-1.31) | 0.85 |
| +Vascular disease | 1.93 (1.85-2.01) | <.0001 | 1.96 (1.85-2.07) | <.0001 |  | 1.02 (0.84-1.23) | 0.87 | 0.98 (0.73-1.33) | 0.91 |
| +Alcohol use | 1.94 (1.87-2.02) | <.0001 | 1.95 (1.85-2.06) | <.0001 |  | 1.00 (0.83-1.21) | 0.99 | 0.97 (0.72-1.31) | 0.86 |
| +Smoking status | 1.91 (1.83-1.98) | <.0001 | 1.86 (1.76-1.96) | <.0001 |  | 1.02 (0.85-1.23) | 0.82 | 0.99 (0.73-1.33) | 0.92 |
| +ACE inhibitor use | 1.92 (1.85-2.00) | <.0001 | 1.96 (1.86-2.08) | <.0001 |  | 1.02 (0.85-1.23) | 0.84 | 0.98 (0.73-1.32) | 0.91 |
| +ARB use | 1.92 (1.85-2.00) | <.0001 | 1.96 (1.86-2.07) | <.0001 |  | 1.01 (0.84-1.22) | 0.88 | 0.97 (0.72-1.31) | 0.87 |
| **Fully adjusted** | 1.93 (1.85-2.01) | <.0001 | 1.84 (1.74-1.94) | <.0001 |  | 0.97 (0.80-1.17) | 0.74 | 0.99 (0.73-1.34) | 0.94 |
| Abbreviations: Covid-19, coronavirus disease 2019; OR, odds ratio; CI, confidence interval; COPD, chronic obstructive pulmonary disease; ACE-inhibitor, angiotensin converting enzyme inhibitor; ARB, angiotensin II receptor blocker | | | | | | | | | |
| Note: Age-adjusted models include race/ethnicity and age. Demographic-adjusted models additionally include sex, rural/urban residence, and condition on site of care. Fully adjusted models include all variables and condition on site of care. Individual adjustments made between these three primary models are not inclusive of other adjustments made. | | | | | | | | | |
